# Effects of Wheat Tempering with Slightly Acidic Electrolyzed Water on the Microbiota and Flour Characteristics

**DOI:** 10.3390/foods11243990

**Published:** 2022-12-09

**Authors:** Mingqian Qin, Yingwu Fu, Ning Li, Yinyin Zhao, Baowei Yang, Li Wang, Shaohui Ouyang

**Affiliations:** 1 College of Food Science and Engineering, Northwest A&F University, Yangling, Xianyang 712100, China; 2 Shenzhen Research Institute, Northwest A&F University, Shenzhen 518000, China; 3 Engineering Research Center of Grain and Oil Functionalized Processing in Universities of Shaanxi Province, Yangling, Xianyang 712100, China

**Keywords:** wheat tempering, grain processing, high throughput sequencing, antimicrobial efficacy, physicochemical properties, slightly acidic electrolyzed water (SAEW)

## Abstract

Slightly acidic electrolyzed water (SAEW) was prepared and used as wheat tempering water. This study explored the impacts of tempering with SAEW on microbial load and diversity and quality properties of wheat flour. As SAEW volume ratio increased, the residual level of total plate counts (TPC) and mould/yeast counts (MYC) decreased dramatically (*p* < 0.05). Based on genomics analysis, bacterial 16S rRNA gene and fungal ITS1 gene region were performed to characterize the changes in microbial communities’ composition and diversity in response to SAEW treatment. SAEW optimal volume ratio (6.5:10, *v*/*v*) of SAEW with distilled water influenced wheat microbiome composition, with a higher microbial diversity and abundance discovered on the control grains. *Bacteroidetes* of predominant bacterial phylum and *Ascomycota* of the most abundant fungal phylum were reduced after SAEW optimal volume ratio tempering. The flour yield is higher and ash content is lower than the control samples. Falling number and “b*” in terms of colour markedly increased. DSC (Differential Scanning Calorimetry) test showed that To (onset temperature), Tp (peak temperature), and Tc (conclusion temperature) were significantly decreased in thermal characteristics of flour. Gluten content, protein content, ΔH and pasting properties tests showed no significant change. It can be concluded that SAEW should be applied on wheat tempering for producing clean wheat flour. ANOVA and Tukey’s honestly significant difference (HSD) test were used for the analysis of variance and differences between the experimental and control groups, with *p* < 0.05.

## 1. Introduction

Wheat represents the most commonly used cereal grain for human diets and consumption [1]. However, wheat grains are inevitably contaminated by microorganisms in the growing, drying, storage, and transportation environment, in which bacteria represents the largest number of microbial species, followed by yeast and moulds [2]. The grain is a potential carrier for the development of microbial colonies growing and reproducing as a result of wheat being rich in nutrients. Berghofer [3] studied the distribution of microorganisms in Australian wheat and found that the most common flora was Bacillus spp. (10^4^ cfu/g), followed by coliform (10 MPN/g), yeast (10^3^ cfu/g), and mould (10^3^ cfu/g). The most common moulds isolated were *Aspergillus*, *Penicillium, Cladosporium*, and *Eurotium* spp. Xiang [4] (pp. 205–211) conducted a comprehensive investigation on the representative wheat microflora in the eight major wheat regions in China and found 30 genera, 101 species of mould, and 3 genera of yeast. The bacterial colony carried in raw wheat can reduce the nutritional value and cause material loss and off-odours, leading to poor flour quality, which can be harmful to humans and animals [5]. Thus, wheat kernels with high microbial loads lead to the degeneration of flour and shorten the shelf-life of flour-based products, especially fresh wet noodle products. There are some problems that nutritional indicators have highlighted, while the microbial indicators have not been paid much close attention.

The microflora of wheat kernels is abundant and complex and mainly originates from various stages of wheat processing. Wheat tempering is a necessary processing stage before milling to improve the plasticity of the outer bran layer, to prevent it from fracturing, and ensure easier separation from the endosperm [6]. However, the long time, appropriate temperature, and proper humidity of tempering are conducive to the growth of bacteria and fungus on the surface of the wheat kernels. Once these microorganisms—especially food-borne pathogens—contaminate wheat flour, they will survive for significant periods under ambient storage conditions [7]. With the continuous upgrading of social consumption and knowledge around health, consumers and downstream areas of the flour industries have put forward low-bacteria requirements for raw wheat and flour.

SAEW is a novel non-thermal sterilizing agent produced by the electrolysis of dilute hydrochloric acid or sodium chloride solution, or a mixed solution of both of them, in a direct current electrolytic device [8]. Available chlorine concentration (ACC) of 10–30 mg/L, pH value of 5.5 to 6.5 for SAEW is widely used as a disinfectant to reduce the microbial load. The antibacterial mechanism of SAEW is due to the presence of available chlorine and high redox potential. Chloride ions produced by available chlorine (mainly hypochlorite) can easily penetrate into bacteria and alter the osmotic pressure of microbial cells, causing them to become inactive or even die. Oxidation reactions are responsible for the deleterious effects on the important enzymes and thus interfere with bacterial metabolic activity [9]. It is reported that SAEW has been extensively applied to inactivate or inhibit various food-borne pathogens on seafood, ready-to-eat vegetables, pasta, meat, and so on [10,11,12]. Additionally, SAEW has been regarded as a legal food additive in Japan since 2002 [13]. Therefore, it has become a popular alternative to traditional chlorinated chemical sterilizer based on its toxin-free, non-axenic, eco-friendly, and biologically safe nature.

In certain factories, pure water is usually used to temper wheat. This work aimed to evaluate the disinfection efficacy of tempering with SAEW on microbial load and microflora diversity. The population levels of total plate colonies, mould, and yeast were determined after different volume ratios (SAEW: distilled water, *v*/*v*, 5:10, 5.5:10, 6:10, 6.5:10 and 7:10) of SAEW tempering treatment. Attention was focused on reducing the natural microbial flora carried on the grain surface, since contamination typically occurs before and after processing. To characterize the composition of the fungal and bacterial communities in grains of an optimal volume ratio (6.5:10), SAEW and distilled water tempering treatments, amplicon-based high-throughput sequencing (HTS) of 16S rRNA, and internal transcribed spacer (ITS) were performed. At the same time, the quality properties of flour were also evaluated.

## 2. Materials and Methods

### 2.1. Materials and Chemicals

The Weilong-169 wheat (Y), a kind of semi winter medium mature variety (12.02 g protein, 1.48 g total ash per 100 g whole wheat flour; hardness index 66.53) was harvested and obtained from Jinshahe flour production Corporation (Wugong, China) in September 2021, and its moisture content was measured as 11.08%. The wheat grains were cleaned by hands and sieves. An amount of 37% hydrochloric acid (HCl), solid sodium chloride (NaCl), concentrated sulfuric acid (H_2_SO_4_), sodium hydroxide (NaOH), and boric acid (H_3_BO_3_) of analytical grades were used, and were purchased from XinRuitai chemical supply station (Yangling, China).

### 2.2. SAEW Preparation

SAEW was prepared using an Electrolyzed Water Generation device (Harmony-Ⅱ, Ruiande environmental equipment Company Ltd., Beijng, China). The electrolytic current was set as 19.2 A. Distilled water was used to prepare 50 mL electrolyte solution composed of 10% NaCl and 1% HCl solutions [14]. The electrolyte solution and 4 L distilled water were pumped into SAEW generator together and electrolyzed for 8.5 min to obtain SAEW. Original SAEW with the pH value of 2.66, ACC of 93 mg/L and Oxidation–Reduction Potential (ORP) of 1068 mV was produced in a cylindrical vessel. The original SAEW and distilled water were mixed at a volume ratio of 7:10, 6.5:10, 6:10, 5.5:10 and 5:10 to obtain SAEW with different ACC determined by a digital chlorine kit (Chlorometer Duo, Palintest Co., Gateshead, UK). The pH and ORP values of the disinfectants were measured using a dual scale pH/ORP meter (model KP-10z, Kasahara Chemical instruments Corp., Tokyo, Japan) bearing a pH electrode and an ORP electrode. SAEW was required to be measured and used immediately after preparation. Table 1 shows the detailed information of SAEW.

### 2.3. Wheat Tempering

An amount of 500 g of cleaned wheat grain was put into a sterile plastic bag. The target moisture content of the wheat kernels before grinding was adjusted to 15.0% by adding SAEW and then was shook uniformly. After addition of the solution, sterile plastic bags containing grains were sealed and balanced at room temperature (18 °C) for 24 h. The volume required for the solution could be calculated using the following equation:

(1) $$V=\frac{M(W_{1}-W_{0})}{\left(1-W_{1}\right)}$$

where *M* (g) marks the weight of wheat sample, namely 500 g. *W*_1_ (%) shows the target moisture content of wheat grains after tempering, namely 15%. *W*_0_ (%) represents the initial moisture content of wheat grains before milling, namely 11.08%. The control group as well as the traditional wheat tempering group was tempered with distilled water for 24 h at the same temperature and other conditions.

### 2.4. Grains Grinding

The tempered wheat samples were milled using a Quadrumat Jr laboratory Mill (Brabender^®^, Duisburg, Germany) under laboratory conditions. Wheat grains were completely ground into bran and flour. The flour yield was calculated as the ratio of the weight of flour to the weight of flour and bran.

### 2.5. Analysis of Microbial Concentrations

An amount of 25 g of wheat kernel tempered by each SAEW volume ratio solution was aseptically transferred into 225 mL 0.85% sterilized physiological saline solution in a sterile ziplock bag and mixed manually until well combined. The microbial amount of wheat grains after tempering adjustment was determined using nutrient agar medium. The viable count was performed using the standard plate count method according to [15]. Sterilized physiological saline solution was also used to finish serial ten-fold dilutions. An amount of 1 mL of the appropriate dilution was dropped onto sterile Plate Count Agar (PCA) (Land Bridge, Beijing land bridge technology Co., Ltd., Beijing, China) and cultured at 37 °C for 48 h to enumerate. For yeast and mould counts, 1 mL diluted solution was poured into Bengal Rose Agar (Land Bridge) and incubated at 25 °C for 120 h [16].

### 2.6. Amplicon-Based Metagenomic Analysis

According to the preliminary wheat tempering process optimization results of our laboratory studies, the volume ratio of SAEW was 6.5:10, tempering time 21 h, and the target water content 14.75%, resulting in higher flour yield and better quality. Microbial diversity analysis experiments were conducted on this basis.

Filter: A total of 30–40 g of wheat grain sample was selected and tempered with SAEW under the optimal conditions. The surface of the grains was flushed and filtered through a 0.22 μm membrane with 1 L sterilized distilled water. The filter membrane containing microorganism filtrate was collected in a 5 mL centrifugal tube and then used for isolation of microbial genomic DNA, which were then delivered to the sequencing company for detection.

Amplicon Generation: The 16S rRNA and internal transcribed spacer (ITS) of distinct regions 16S V3–V4, and ITS1, respectively, were amplified using specific primers: bacterial 16S 338F (5′-ACTCCTACGGGAGGCAGCA-3′) and 806R (5′-GGACTACHVGGGTWTCTAAT-3′), fungal ITS ITS5F (5′-GGAAGTAAAAGTCGTAACAAGG-3′) and ITS1R (5′-GCTGCGTTCTTCATCGATGC-3′), to analyse several bacterial and fungal taxa. All PCR reactions were carried out with TransStart^®^ FastPfu DNA Polymerase. The PCR products were then used to verify amplification and detected by 1.2% agarose gel electrophoresis.

Illumina MiSeq sequencing and data processing: Sequencing libraries were prepared using a TruSeq Nano DNA LT Library Prep Kit (Illumina Inc., San Diego, CA, USA). The libraries were checked before sequencing and qualified libraries with single peak and no connector were selected to carry out high throughput sequencing. Sequence stitching, identification and trim, and quality control were carried out according to the analysis process of Vsearch software [17]. For each representative sequence, operational taxonomic unit (OTU) clusters and taxonomic annotation were implemented, which were used for further analysis. All the sequences clustered into OTUs at 97% similarity level were considered to be homologous in species [18].

### 2.7. Effects of Optimal SAEW Tempering on Flour Characteristics

#### 2.7.1. Compositional and Physicochemical Analysis

The flours from wheat grains tempered by optimal volume ratio SAEW and traditional distilled water were analysed for ash content as reported by Mirani [19], crude protein content—N × 6.25 was measured by the Kjeldahl method according to Association of Official Analytical Chemists (1984) procedures [20]. Falling number was determined using the method described by AACC Method 56–81.03 [21]. The colour characteristics of the flour were measured on Minolta CR300 colorimeter (Minolta Corp., Ramsey, NJ, USA) [22]. Wet gluten and dry gluten content were determined by the hand washing and oven drying method, respectively, according to China National Standards GB/T 5506.1-2008, GB/T 5506.3-2008 [23,24].

#### 2.7.2. Pasting Properties

The pasting properties were measured using a Rapid Visco Analyzer (RVA; TechMaster, Perten, Sweden). Flour sample (3 g) and distilled water (25 mL) (adjusted by 14% wet basis) were weighed and mixed in an aluminium cylindrical canister. The mixture was heated while stirring from 48 °C to 95 °C at a rate of 12 °C/min and 160 r/min. When the temperature rose to 95 °C, it was maintained for 2.5 min and then gradually cooled to 50 °C at the same rate [25].

#### 2.7.3. Thermal Properties

The thermal properties of the wheat flour samples were characterized using a Differential Scanning Calorimetry (DSC Q2000; TA Instrument, Newcastle, DE, USA) analyser including a liquid nitrogen cooling system. Flour sample of 3 mg and 9 μL deionized water were accurately weighted into an aluminium pan and then equilibrated at 4 °C for 12 h. The pan containing sample was sealed hermetically and heated together with an empty reference pan from 20–130 °C at 20 °C/min [26].

### 2.8. Statistical Analysis

All experiments and measurements were conducted in independent triplicate to ensure the reliability of the results [27]. The results were reported as mean ± standard deviation (SD). Data were statistically analysed by one-way ANOVA and Tukey’s honestly significant difference (HSD) tests using Minitab version 18 (Minitab Inc., Statecolidge, PA, USA). Statistical significances were established at a level of *p* < 0.05.

## 3. Results

### 3.1. Microbial Counts in Tempered Wheat Kernels

**Figure 1:**
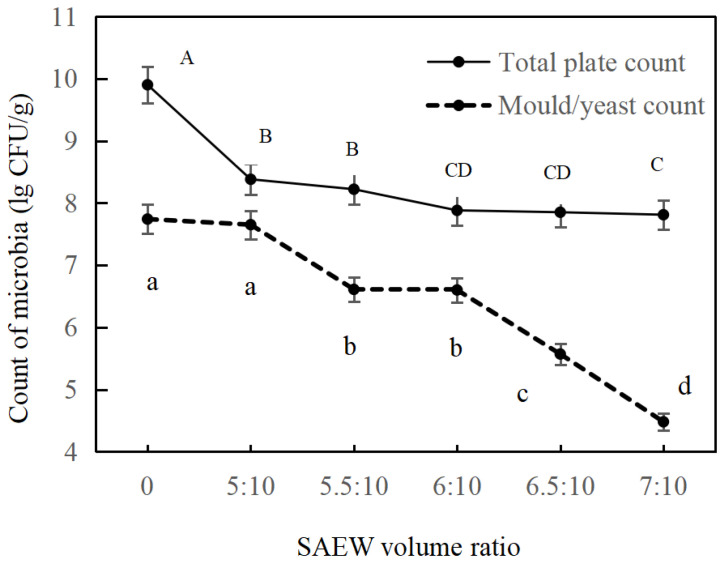
Effect of different volume ratio SAEW on the count of microbes in wheat kernels. The different capital letters (A–D) indicate significant differences within the total plate count; different lowercase letters (a–d) indicate significant differences (*p* < 0.05) across the mould and yeast count; and 0 represents the distilled water tempering group of the control without the addition of electrolytic water. Data points for wheat tempering samples with different volume ratio SAEW are shown as mean ± SD. The error bars are derived from the standard deviation of replicates (*n* = 3).

Mould or yeast count (MYC) can be used as indicators of food hygiene quality. The total plate count (TPC) in wheat grains represents the degree of microbial contamination. Distinctively, the content of MYC and TPC in distilled water tempering grains and in different volume ratios SAEW wheat tempering grains were shown in Figure 1. The initial load of TPC and MYC in distilled water tempering wheat kernels was separately 9.90 lg CFU/g and 7.74 lg CFU/g. The microbial counts in the raw wheat sample were higher than in a study reported by Cardoso et al. [28]. This may be due to the fact that the harvested wheat was not cleaned adequately. With the increase in SAEW volume ratio, both MYC and TPC were decreased significantly (*p* < 0.05) in all samples. Higher SAEW volume ratio induced a larger obvious effect of reducing microbes. The surviving population of TPC in wheat grains was 8.38, 8.22, 7.88, 7.85, 7.81 lg CFU/g after tempering with SAEW at a volume ratio of 5:10, 5.5:10, 6:10, 6.5:10, and 7:10, respectively, while 7.65, 6.61, 6.60, 5.57, and 4.48 lg CFU/g was observed for MYC in wheat grains tempered with SAEW at the same volume ratio. The results were in agreement with the study published by Zhang [29], in which SAEW was used to disinfect total aerobic bacteria, yeast, and mould on celery and cilantro, and found that the total count of these two kinds of microorganism was decreased while the ACC of SAEW increased. As was shown in Figure 1, the TPC of tempered wheat grains after tempering with volume ratio 7:10 of SAEW were reduced by 2.09 lg CFU/g. Meanwhile, approximately 3.26 lg CFU/g of YMC were inactivated in tempered wheat grains. The data indicated that SAEW tempering treatment decreased YMC count remarkably, while TPC was reduced less. The reason that TPC is more resistant to SAEW than YMC, relatively. In addition, complicated organic matters in the outer layers of wheat grains may react with SAEW to protect TPC from destruction [30]. However, compared to previous studies [31], the decreases in TPC and YMC at similar SAEW treatment levels were relatively larger. It could be that initial population of TPC and TMC is higher than that observed by Chen [31]. Finally, the anti-micro-organisms effect of SAEW is significant and effective.

### 3.2. Effects of Optimal Volume Ratio SAEW Tempering on Microbial Community Composition

As many species in the microbial communities are uncultured [32], and the culture method only quantitatively characterized the effect of SAEW on reducing microorganisms, relatively little is known about specific information on microbial species. In order to better explain changes in microbial diversity in a comprehensive manner after SAEW tempering of wheat, we adopted the high-throughput sequencing approach to investigate the abundance, distribution, diversity, and composition of microbial flora.

#### 3.2.1. Relative Abundance

**Figure 2:**
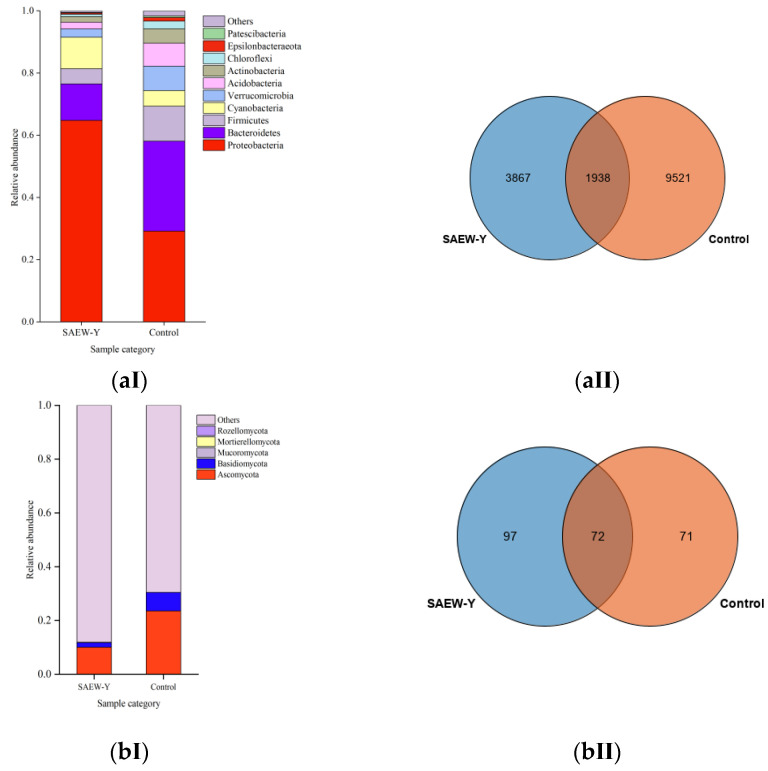
Taxa relative abundance in phylum level for (**aⅠ**) bacterial 16S and (**bⅠ**) fungal ITS regions. Venn diagrams showing the distribution (97% similarity) of the OTUs at genus level present in (**aⅡ**) for bacteria and (**bⅡ**) for fungi. “Others” represents a total relative abundance of the remaining phyla besides the top 10 phyla. Control- tempering weilong-169 wheat grains (Y) with distilled water, SAEW-Y- tempering weilong-169 wheat grains with SAEW, optimal volume ratio 6.5:10, respectively, at the same conditions.

16S and ITS sequencing obtained a large number of effective sequences. Each sample was analysed in triplicates representing the groups by an average read. As is shown in the distribution histogram of relative abundance of taxa from Figure 2, the top 10 taxa of each sample were selected to display their proportion in phylum level. From the identified phyla in the control wheat sample, *Bacteroidetes* (29.16%), *Proteobacteria* (28.93%), *Firmicutes* (11.27%), *Cyanobacteria* (5.02%), *Verrucomicrobia* (7.83%), and *Acidobacteria* (7.37%) constituted the predominant bacterial microbiota (Figure 2a(Ⅰ)). *Bacteroidetes* was the most abundant phylum and decreased to 11.73% after SAEW tempering (Figure 2a(Ⅰ)). *Bacteroidetes* mainly included *Bacteroidia* (86.41%), *Chlorobia* (0.28%), and *Ignavibacteria* (0.12%) detected in control samples. After SAEW tempering treatment, the dominance of *Bacteroidia* decreased to 35.03% and *Chlorobia* reduced to 0.08%. A small proportion of sequences were assigned to groups *Chloroflexi* (2.52%), *Epsilonbacteraeota* (1.11%), and *Patescibacteria* (0.47%). The abundance of the above phyla decreased to 0.75%, 0.42%, and 0.17%, respectively, while the dominance of *Proteobacteria* increased proportionally. A higher proportion of fungal regions is closely related to the ITS method. ITS sequencing identified a total of six recognized phyla and the most abundant fungal phylum was *Ascomycota*, followed by *Basidiomycota*, and *Mucoromycota* (Figure 2b(Ⅰ)). These categories are phytopathogens capable of infecting plants, including wheat, which cause spoilage in stored grains and expose human and animal health to extreme danger [33,34]. As shown in Figure 2b(Ⅰ), SAEW-Y grains samples had a lower relative abundance for *Ascomycota* (10%) and *Basidiomycota* (1.8%) compared with the control grains (23.5/6.8%). The phylum *Mortierellomycota* performed at a lower level, but with similar abundance in both groups (<0.01% and 0.02%) between SAEW tempered and control samples detected with ITS. Some fungal genera, for example *Rhodotorula* of *Basidiomycota* and *Gibberella*, *Talaromyces* of *Ascomycota* were less abundant or not detected after SAEW treatment. The phylum that others detected with ITS reads covered unclassified fungi and unidentified species, which cannot yet be classified based on current available taxonomical reference data [35]. They were closely related to endophytes of wheat and further analysed for host species. The changes in the microflora between different tempering treatments revealed the effect of SAEW applied on the bacterial and fungal community.

OTU numbers express the sample species richness [36]. There are 5805 OTUs in the SAEW tempering group, and 11,459 OTUs in the control treatment group. Both of them shared 1938 OTUs (Figure 2a(Ⅱ)). The results showed that the bacterial species composition of SAEW-Y and control sample were quite different. Similarly, fungal OTUs distribution in SAEW-Y and control sample were 169 and 143, respectively (Figure 2b(Ⅱ)). The 72 OTUs are both jointly owned by them, illustrating no difference in the composition of major fungal species.

#### 3.2.2. Taxonomic Abundance Cluster Heatmap

**Figure 3:**
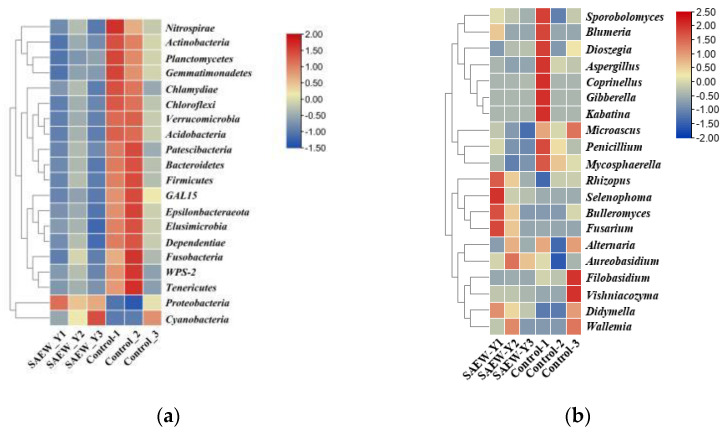
A hierarchically clustered heatmap of the microbial profiles of SAEW and control grains samples based on (**a**) Greengenes database and (**b**) UNITE database taxonomy assigned to genus levels. The relative abundance of the bacterial (**a**) and fungal (**b**) groups were log 2 transformed. The *X*-axis and the *Y*-axis represent sample name and the genus, separately. SAEW-Y1, SAEW-Y2, and SAEW-Y3 are the parallel treatment groups of SAEW, and the same for control.

Based on abundance information of the top 20 phyla/genera in the samples, heatmaps were drawn, which visualized the similarities and differences in the microbial communities’ structure of SAEW and control tempering wheat in the samples [37]. The phylum-level and genus-level heatmaps separately showing the bacterial (a) and fungal (b) profiles from both tempering groups are clearly different. As Figure 3 shows, there is a wide diversity of bacterial and fungal communities and species richness in control samples. *Bacteroidetes*, *Firmicutes*, *Epsilonbacteraeota*, and *Chloroflexi* belonging to bacterial phyla were richer in the control groups (Figure 3a). Similarly, *Filobasidium*, *Penicillium*, *Sporobolomyces*, *Aspergillus*, and *Microascus* originated from the ITS regions, and a larger proportion of more abundant fungal genera were surveyed on control groups than on SAEW groups (Figure 3b). Moreover, SAEW treatment samples contained a low number of *Gibberella* and *Kabatina*, which were even not detected compared to the control samples. The result is consistent with the relative abundance report as shown in Figure 2a,b.

#### 3.2.3. The Alpha and Beta Diversity Indices of the Microbial Community

**Figure 4:**
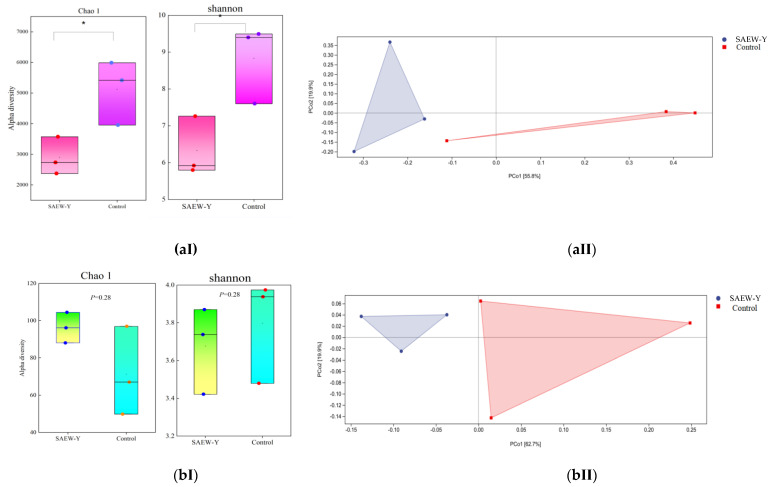
Boxplots of Alpha diversity indices and PCoA analysis based on Bray-Curtis distances between the microbial communities presented in the SAEW and control tempering wheat grains samples. (**a**), bacterial 16S sequencing; (**b**), fungal ITS regions sequencing. Diversity richness (Chao1) and diversity indices (Shannon) of bacteria (**aⅠ**) and fungi (**bⅠ**). PCoA analysis of Beta diversity for bacteria (**aⅡ**) and fungi (**bⅡ**). Each point represents an individual sample. * indicate significant differences (*p* < 0.05).

From Figure 4a, bacterial α- and β- diversity identified significant differences among those SAEW tempering groups compared with those assigned to the control groups at genus level. Chao1 and Shannon indices showed that bacterial diversity varied significantly (*p* < 0.05) between SAEW-Y and control samples. These two indices responding to the number of observed bacterial species were always higher for the control sample. The marked decrease in Chao1 and Shannon indexes after SAEW treatment indicated the decline in the initial microflora in the tempered wheat sample (Figure 4a(Ⅰ)). In contrast, as observed with ITS gene sequencing, the Chao1 index was higher and Shannon index lower than the control group after SAEW tempering treatment, indicating no significant difference (*p* ≥ 0.1) in fungal community composition between the two groups. (Figure 4b(Ⅰ)). This is mainly because of the endophytic flora of plants disturbing the classification of fungi. Certainly, the number of unique fungal species on the SAEW-tempered samples were lower than that of control. The result illustrated that it corresponded to the Venn diagram (Figure 2b(Ⅰ),b(Ⅱ)). The PCoA diagrams based on Bray-Curtis dissimilarity metric were constructed. The Pco1 and Pco2 axes showed values of cumulative percentage variance of species equal to (55.8% and 19.9%) for bacteria and (62.7% and 19.9%) for fungi, respectively. It was revealed in Figure 4a(Ⅱ),b(Ⅱ) that microbial communities clustered to SAEW tempering samples, while control groups were relatively dispersed.

### 3.3. Physical and Chemical Properties of Flour

Decontamination to gain clear flour is one of the main goals. On the other hand, it is also very important to ensure the quality of flour after SAEW wheat tempering. Physical and chemical properties of optimal volume ratio SAEW and control wheat tempering samples are provided in Table 2. It observed a lower level of ash content (0.53 ± 0.01%) and a higher level of flour yield (73.3 ± 0.1%) in the SAEW sample. The result presented a similar value to the study reported by Şenol İbanoǧlu [38]. Ash content correlates well with the yield of flour. High flour yield but low ash content naturally are desirable results. Therefore, SAEW is to the benefit of high fineness flour production. In this study, no significant differences were observed in the gluten and protein content of SAEW wheat tempering sample compared to the control one. This means that suitable SAEW has little effect on the dough and baking properties of flour. Falling number was found to increase significantly (from 337 ± 20 s to 379 ± 6 s) after SAEW tempering. The value indicated that the enzyme activity in the grains was weakened because the falling number has a negative connection with α-amylase activity. However, excessive α-amylase activity easily leads to the production of sticky doughs that are difficult to process. Different flour products need their own moderate falling number. After SAEW wheat tempering, the falling number is 379 ± 6 s, which is a reasonable range for Chinese steamed bread production [39]. In addition, colorimetric parameters of the “L*” and “a*” values did not change significantly. This means that SAEW tempering had little effect on the lightness and redness of flour. Notably, the “b*” value of SAEW-Y sample (8.76 ± 0.32) had a remarkable increase compared to the control (7.82 ± 0.01). It is possible that the SAEW pH environment or free ions play a role in the natural pigments (such as carotene) causing the change. Pigment can be studied in depth by extraction technology and spectrometry for further analysis. The combination of high oxidation potential and available chlorine of SAEW is the main reason for the changes in the physicochemical properties of flour [40].

### 3.4. Pasting Properties

**Figure 5:**
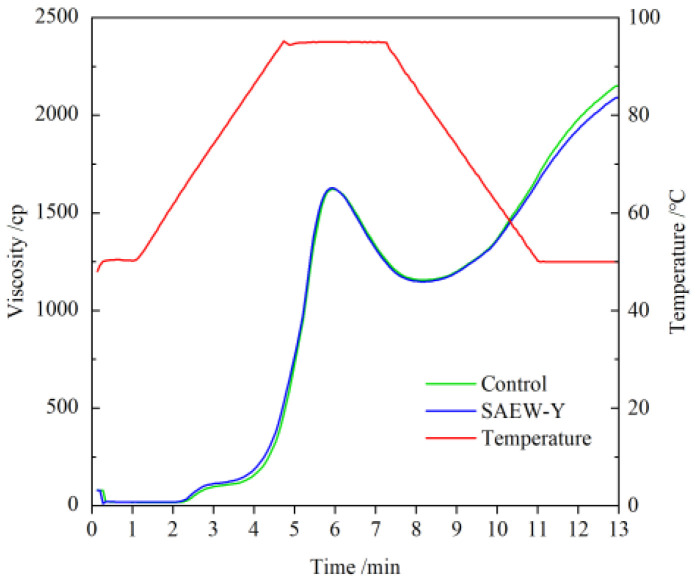
Pasting properties of control and SAEW tempering flour samples analysed using a Rapid Visco-Analyzer (RVA).

The pasting properties of wheat flour after different tempering treatments during heating and cooling are shown in Figure 5. The viscosity parameters are summarized in Table 3. There was no obvious difference in the peak pattern and peak time of pasting properties curves after tempering. Compared with the PV and TV of control flour (1623 ± 23 cP, 1157 ± 11 cP), SAEW tempering treatment had no significant effect on them, presenting 1628 ± 12 cP and 1149 ± 0 cP, respectively. Hence, the BD of both (466 ± 12 cP, 479 ± 12 cP) also shows no obvious difference. In addition, they reach peak viscosity almost at the same time (namely, peak time-PET). Adversely, the FV, SB, and PT of SAEW-Y flour show inconsistent changes compared with that of the control flour. SAEW-Y flour displayed lower FV and PT (2092 ± 13 cP and 88.45 ± 0.01 °C) than the control flour (2152 ± 25 cP and 89.65 ± 0.06 °C). SAEW carries negative ions and mutually repels. It will strengthen the swelling of starch particles and cause SAEW-Y flour to exhibit a lower pasting temperature [41]. In addition, the lower SB (943 ± 13 cP) observed after SAEW tempering may result from the weak interaction between amylose and other starch molecules during cooling, which is beneficial for improving the anti-retrogradation capability to a certain extent.

### 3.5. Thermal Characteristics

**Figure 6:**
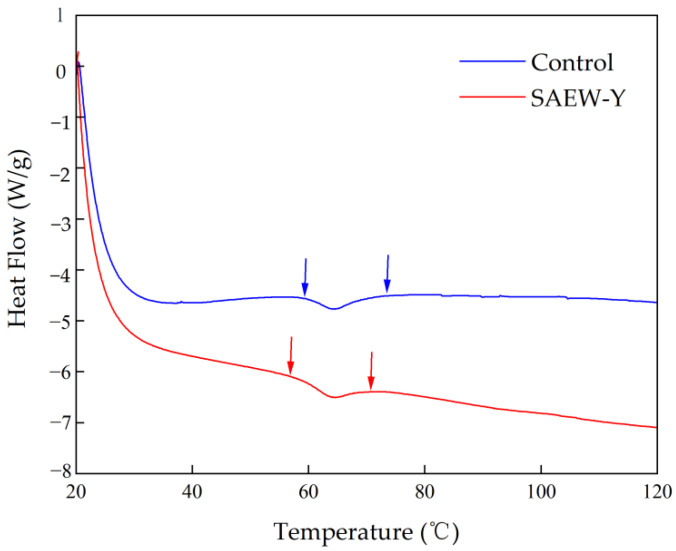
DSC profiles of different wheat tempering flour samples. Tempering wheat with distilled water (control) and tempering wheat with SAEW (SAEW-Y) flour samples at the same moisture content of 14% are shown.

Starch is an essential ingredient in cereal foods and is mainly composed of amylose and amylopectin. It can be seen from Figure 6 that the DSC thermograms of different wheat tempering samples are shown with the To and Tc indicated by arrows on the graph. Both control and SAEW-Y groups displayed a single clear melting endothermic peak of crystal structure from the water and starch molecular chain interaction during heating. The thermal transformation parameters involving onset temperature (To), peak temperature (Tp), conclusion temperature (Tc), and enthalpy change (ΔH) are recorded in Table 3. The To, Tp, and Tc (60.19 ± 0.05 °C, 64.20 ± 0 °C and 73.77 ± 0.45 J/g) in the control sample were decreased significantly to 58.98 ± 0.07 °C, 63.11 ± 0 °C, and 69.27 ± 0.16 J/g after SAEW tempering, respectively. Meanwhile, it showed a mildly higher gelatinization enthalpy (ΔH), but no significant difference was observed. SAEW treatment decreased the melting temperature, mainly due to enhancing the role of amorphous regions [42]. An increase in the homogeneity of starch crystallites and increase in the synergistic melting between the crystalline and amorphous regions would lead to the same results [43]. Meanwhile, SAEW weakened the hydrogen bonding between starch molecules to decrease the energy required for starch gelatinization [44]. Some minor components of starch granules and granules size also remarkably affect the thermal properties. Thus, the lower gelatinization enthalpy was explained in SAEW-Y flour. Further research on molecular structure in flour tempered with SAEW will be carried out.

Based on the above results, SAEW optimal volume ratio (SAEW:distilled water, 6.5:10) was recommended as a wheat tempering processing condition. It is necessary to point out that the processing time and volume added should be adjusted according to the wheat varieties. SAEW tempering changed some qualities of the flour (for example, the thermal quality, falling number, and “b” value), but there was no seriously adverse effect on the flour. The underlying reasons and connections for the difference will be reported later in another manuscript. As for pasting properties, i.e., protein, gluten, chrominance (L* and a*), and so on, no significant differences were detected. Therefore, SAEW can be applied prior to milling to address the various risks of microbial contamination of wheat grain for food safety.

## 4. Conclusions

This study demonstrates that SAEW is a potentially useful reagent for wheat grain decontamination and clean flour production. The MYC and TPC decreased significantly (*p* < 0.05), resulting in an inactivation level of up to 3.26 lg CFU/g and 2.09 lg CFU/g, respectively, in wheat grains tempered by SAEW. As SAEW volume ratio increases, ORP and ACC of SAEW significantly varied. They could collectively contribute to the antimicrobial capacity of SAEW. Simultaneously, SAEW tempering had a significant impact on the structure and composition of the wheat microbiome. Higher microbial diversity and more complicated composition were discovered on the control samples as compared to SAEW-treated grain samples. SAEW tempering was effective against a range of bacteria and fungi on the surface of wheat grains. Further studies are necessary to understand the mechanisms of inactivation of certain microflora mediated by SAEW. It is worth noting that SAEW is not a one-size-fits-all solution, where microbiome reductions must be considered in combination with management systems in wheat processing circuits. Meanwhile, the pasting stability and protein and gluten content of flour showed no significant change (*p* > 0.05), while the onset temperature, peak temperature, and conclusion temperature of thermal property in flour were significantly decreased as they were affected by SAEW (*p* < 0.05). Overall, SAEW wheat tempering can construct an important line of defence for wheat flour cleaning production.

## Figures and Tables

**Table 1:** Specific parameters of slightly acid electrolyzed water.

SAEW Volume Ratio (*v*/*v*)	pH Value	ORP/mv	ACC/mg/L
5:10	6.06 ± 0.02 ^a^	890 ± 5 ^a^	9 ± 2 ^e^
5.5:10	6.02 ± 0.01 ^ab^	886 ± 2 ^a^	15 ± 2 ^d^
6:10	5.98 ± 0.02 ^bc^	872 ± 4 ^b^	20 ± 2 ^c^
6.5:10	5.88 ± 0.01 ^cd^	861± 3 ^c^	25 ± 2 ^b^
7:10	5.83 ± 0.02 ^d^	860 ± 3 ^c^	32 ± 1 ^a^

Data points for different volume ratio of SAEW were presented as means of triplicate assays ± standard deviation. Values with different letters in the same column were significantly different among SAEW solutions detected by Tukey’s test (HSD, ANOVA, *p* < 0.05). In the table, SAEW represents slightly acidic electrolyzed water, ORP expresses oxidation–reduction potential, and ACC indicates available chlorine concentration.

**Table 2:** Evaluation of physical and chemical quality of wheat flour tempered by distilled water (control) and SAEW.

Sample	Properties								
	Ash Content (%)	Flour Yield (%)	Gluten Content (%)		Protein (%)	Falling Number (s)	Chromaticity		
			Wet Gluten	Dry Gluten			L*	a*	b*
Control	0.59 ± 0.03 ^a^	72.6 ± 0.1 ^a^	36.48 ± 0.44 ^a^	11.49 ± 0.12 ^a^	11.79 ± 0.41 ^a^	337 ± 20 ^b^	89.88 ± 0.01 ^a^	0.57 ± 0.02 ^a^	7.82 ± 0.01 ^b^
SAEW-Y	0.53 ± 0.01 ^a^	73.3 ± 0.1 ^a^	35.11 ± 0.34 ^a^	12.31 ± 0.10 ^a^	11.78 ± 0.29 ^b^	379 ± 6 ^a^	89.63 ± 0.44 ^a^	0.52 ± 0.06 ^a^	8.76 ± 0.32 ^a^

All values are mean of triplicate determinations ± SD. Values followed by the different letter in the same column are significantly different (*p* < 0.05). (L* =lightness, a* = redness, b* = yellowness of wheat flour after tempering).

**Table 3:** Pasting parameters and thermal properties of the control and SAEW tempering flour samples.

Sample	Pasting Properties							Thermal Properties			
	PV/cP	TV/cP	BD/cP	FV/cP	SB/cP	PET/min	PT/°C	Gelatinization Temperature			
								To/°C	Tp/°C	Tc/°C	ΔH/J/g
Control	1623 ± 23 ^a^	1157 ± 11 ^a^	466 ± 12 ^a^	2152 ± 25 ^a^	995 ± 14 ^a^	5.97 ± 0.02 ^a^	89.65 ± 0.06 ^a^	60.19 ± 0.05 ^a^	64.20 ± 0 ^a^	73.77 ± 0.45 ^a^	4.95 ± 0.06 ^a^
SAEW-Y	1628 ± 12 ^a^	1149 ± 0 ^a^	479 ± 12 ^a^	2092 ± 13 ^a^	943 ± 13 ^a^	6.00 ± 0.05 ^a^	88.45 ± 0.01 ^a^	58.98 ± 0.07 ^b^	63.11 ± 0 ^b^	69.27 ± 0.16 ^b^	5.44 ± 0.16 ^a^

PV, peak viscosity; TV, trough viscosity; BD, breakdown; FV, final viscosity; SB, setback; PET, peak time; PT, pasting temperature. All values are means of triplicate determinations ±SD. Means within columns with different letters are significantly different (*p* < 0.05).

## Data Availability

Data are contained within the article.
